# Ecophysiological and Cell Biological Traits of Benthic Diatoms From Coastal Wetlands of the Southern Baltic Sea

**DOI:** 10.3389/fmicb.2021.642811

**Published:** 2021-04-12

**Authors:** Lara R. Prelle, Martin Albrecht, Ulf Karsten, Pauline Damer, Tabea Giese, Jessica Jähns, Simon Müller, Louisa Schulz, Lennard Viertel, Karin Glaser

**Affiliations:** Applied Ecology and Phycology, Institute of Biological Sciences, University of Rostock, Rostock, Germany

**Keywords:** growth rate, photosynthesis, respiration, salinity, temperature, lipids, peatland, PDMPO

## Abstract

The German Baltic Sea coastline is characterized by sea-land transitions zones, specifically coastal peatlands. Such transition zones exhibit highly fluctuating environmental parameters and dynamic gradients that affect physiological processes of inhabiting organisms such as microphytobenthic communities. In the present study four representative and abundant benthic diatom strains [*Melosira nummuloide*s, *Nitzschia filiformis*, *Planothidium* sp. (st. 1) and *Planothidium* sp. (st.2)] were isolated from a Baltic Sea beach and three peatlands that are irregularly affected by Baltic Sea water intrusion. Ecophysiological and cell biological traits of the strains were investigated for the first time as function of light, temperature and salinity. The four strains exhibited euryhaline growth over a range of 1–39 S_A_, surpassing *in situ* salinity of the respective brackish habitats. Furthermore, they showed eurythermal growth over a temperature range from 5 to 30°C with an optimum temperature between 15 and 20°C. Growth rates did not exhibit any differences between the peatland and Baltic Sea strains. The photosynthetic temperature optimum of the peatland diatom isolates, however, was much higher (20–35°C) compared to the Baltic Sea one (10°C). All strains exhibited light saturation points ranging between 29.8 and 72.6 μmol photons m^–2^ s^–1^. The lipid content did not change in response to the tested abiotic factors. All data point to wide physiological tolerances in these benthic diatoms along the respective sea-land transitions zones. This study could serve as a baseline for future studies on microphytobenthic communities and their key functions, like primary production, under fluctuating environmental stressors along terrestrial-marine gradients.

## Introduction

The Baltic Sea is almost completely surrounded by land masses and the tidal range is small (Jurasinski et al., 2018). Nevertheless, wind and atmospheric pressure can cause strong waves and changes in water levels including storm floods (Lass and Magaard, 1996; Karsten et al., 2012). The lasting sea level rise is strengthened by the isostatic subsidence of the southern Baltic Sea coastline (Johansson et al., 2014), whereby the shoreline continuously recedes over time (Harff et al., 2017). Due to this decline, exchange processes between the Baltic Sea and the proximate land will increase in the future facilitated by sea level rise (Jurasinski et al., 2018). Strong gradients in light climate, temperature and salinity across the sea-land transition zone might be the consequence, which in turn affect all inhabiting biota, for example, benthic microalgae (Karsten et al., 2012).

Wide shallow ecosystems are common for low-lying coastal areas of the southern Baltic Sea (Jurasinski et al., 2018) which are sensitive to sea-level rise. Peatlands, such as the nature reserve “Heiligensee und Hütelmoor” (in brief and in the following “Hütelmoor”), often constitute the sea-land transition zone (Jurasinski et al., 2018). The Hütelmoor is located near Rostock, Mecklenburg-Western Pomerania, north-eastern Germany. Like most peatlands in northern Germany, it has been artificially drained with the intensification of anthropogenic land use in the last centuries (Jurasinski et al., 2018). Today nature conservation has been given priority and the Hütelmoor is part of a restoration project, which aims to recreate the original conditions as a coastal peatland by introduced rewetting. Flood protection measures like dunes, which separate this peatland from the Baltic Sea, are not maintained. In the last years two storm surges occurred leading to a massive Baltic Sea water inflow with subsurface saltwater intrusion into the Hütelmoor caused by a break of the main dune.

Besides this natural rewetting of a coastal peatland, other wetlands in north-east Germany are currently reconstructed by fostering their connection to the adjacent Baltic Sea. The polder Drammendorf on the island Rügen, Germany had been cut off from the lagoon Kubitzer Bodden for over 100 years and was rewetted in November 2019 by a dyke removal. This resulted in an inflow of water from the Kubitzer Bodden due to its lower terrain height (Janssen et al., 2019).

While these active rewetting processes will increase in the future, some peatlands have been part of restoration projects for decades. As part of the nature reserve “Insel Koos, Kooser See und Wampener Riff,” the previously drained coastal peatland “Karrendorfer Wiesen” near Greifswald, Germany has been under a rewetting process since 1993 after a dyke deconstruction (Seiberling et al., 2008). This has led to sporadic and periodical flooding by the Baltic Sea of low-lying areas of the Karrendorfer Wiesen (Janssen et al., 2019).

At all these sites the physico-chemical conditions are drastically changing on short time scales leading to strong gradients and highly dynamic diurnal and seasonal fluctuations, with strong influence on all benthic organisms in the coastal shallow water zone and the adjacent peatlands. Saltwater intrusion due to sea-land exchange processes may influence the biodiversity and distribution as well as the physiological performance of microphytobenthic assemblages by causing sudden and strong changes in salinity. The recent Hütelmoor flooding events created a horizontal salt gradient as salinity increased directly behind the dune with a gradual decline with more inland distance (Jurasinski et al., 2018).

Microphytobenthic communities are typically dominated by mostly pennate diatom species, which strongly contribute to the primary production and act as a filter for nutrients and other fluxes at the water-sediment-interface (Risgaard-Petersen et al., 1994; Falkowski et al., 1998; Cahoon, 1999). Benthic diatoms are vitally involved in the carbon-, nitrogen-, phosphorus- and silicate-cycling in shallow coastal waters (Morel and Price, 2003; Wilhelm et al., 2006). Their biomass is important for carbon, energy and nutrient supply for the marine food web and serve as the main food source for benthic grazers (Cahoon, 1999; Sackett et al., 2016). Therefore, benthic diatoms can be considered as one of the ecologically most important and successful protist groups in shallow coastal waters (Wilhelm et al., 2006).

Growth is the most relevant of all physiological processes in reflecting the fitness of an organism, as it integrates all environmental effects and reflects the acclimation potential. One of the main key features of diatoms is their amorphous silica cell wall, the frustule (Brinkmann et al., 2011). The complex and energy-intensive process of the formation of the frustule can be disrupted by many environmental factors like nutrient deficiency, temperature, and salinity (Davis et al., 2005; Roubeix and Lancelot, 2008; Javaheri et al., 2015; Ma et al., 2019; Petrou et al., 2019). The impact of different abiotic factors, like salinity and temperature, on growth of benthic diatoms from the land-sea interface of the Baltic Sea is almost unstudied so far (Woelfel et al., 2014). Benthic diatoms from the southern Baltic Sea are euryhaline, as they can grow from nearly freshwater up to hypersaline conditions (1–50 S_A_). However, they revealed the highest growth in brackish water (15 S_A_) with rates of 0.8–1.4 μ d^–1^ (Woelfel et al., 2014). Likewise, benthic estuarine diatoms showed a broad tolerance range as they grow in salinities ranging from 9.5 to 32 S_A_ while species isolated from freshwater exhibited impaired growth at higher salinities (Trobajo et al., 2011; Glaser and Karsten, 2020). Similar data on benthic diatoms from the Hütelmoor or other peatlands are missing.

Temperature is another important driver for biotic activity as temperature controls the activity of photosynthetic enzymes (Tcherkez et al., 2006). In the shallow southern Baltic Sea close to the beach temperature seasonally fluctuates from 4°C in winter up to 22°C in summer (Lippert et al., 2017). Previous studies of coastal diatom species showed that most of the investigated species are eurythermal, as there is no or only minor temperature influence on the growth rates between 7 and 27°C (Woelfel et al., 2014). Only very low (4°C) or very high (40°C) temperatures strongly reduced the growth rates of benthic diatoms (Scholz and Liebezeit, 2012). Few studies have been undertaken so far on the influence of temperature on photosynthesis and respiration of benthic diatoms in the Baltic Sea (Woelfel et al., 2014; Prelle et al., 2019). The data of these authors indicate at least 20% photosynthetic efficiency from 5 to 35°C, confirming eurythermal traits.

As a response toward stress, raphid, benthic diatoms can move vertically and horizontally in and on top of sediments (Hillebrand and Sommer, 1997; Wasmund and Uhlig, 2003; Blommaert et al., 2018) by excreting extracellular polymeric substances (EPS) (Beninger et al., 2018).

Diatoms produce and accumulate oil mainly in the form of triacylglycerols (TAGs), which are sequestered in lipid droplets (LDs) in the cytoplasm. The biosynthesis of TAGs is controlled by temperature, light conditions, and salinity (for review see Li-Beisson et al., 2019). Diatoms produce up to 60% of their cellular mass as TAGs (Sheehan et al., 1998). Under favorable conditions only small amounts of these neutral lipids are enriched, while under stress LDs are rapidly formed in high amounts (in general 20–50% of cell dry weight) (Hu et al., 2008). During stress recovery, LDs are remobilized serving as an energy source. Stress leads to an imbalance in energy and redox homeostasis, which must be actively counteracted under energy consumption. In diatoms, lipid accumulation seems to be more relevant as energy rather than as carbon storage (Hu et al., 2008). A large portion of cellular carbon is redirected into TAG metabolism under stressful conditions (Alipanah et al., 2015), leading to an increase in oil synthesis while growth and biomass production are slowed down. Consequently, a higher LD production rate goes ahead with a deprivation in overall productivity of the cell (Sheehan et al., 1998; Meksiarun et al., 2015).

As intact peatlands have been proven to be beneficial as carbon sink and nutrient buffering, renaturation projects have increased along the Baltic Sea coast (Vasander et al., 2003). However, the impact of renaturation and changes of abiotic parameters on microphytobenthic processes have not yet been investigated, despite their strong involvement in ecological processes such as primary production. Here we are investigating the ecophysiological response and cell biological traits of four isolated diatoms strains from the sea-land transition zone to the parameters light availability, temperature, and salinity that highly fluctuating in renaturating peatland systems, addressing the following hypotheses: (I) The peatland diatom strains grow and photosynthesize in a wider temperature range than found in their originating habitat due to their high physiological plasticity. (II) The peatland isolates have low-light requirements for photosynthesis as they are regularly confronted with elevated turbidity. (III) The peatland diatom strains exhibit a broad salinity tolerance because of recurring floods of the Baltic Sea, which cause an increased salinity gradient in the coastal peatlands.

## Materials and Methods

### Study Site

**FIGURE 1:**
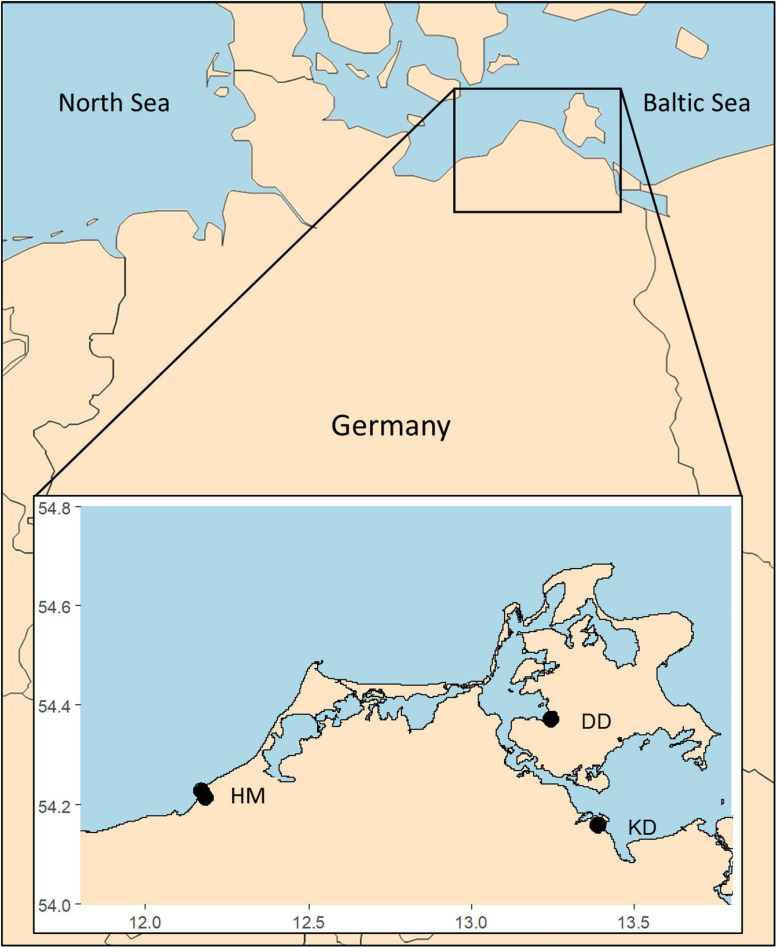
Map of Germany with the respective sampling sites marked with black dots, HM, Hütelmoor, DD, Drammendorf, KD, Karrendorf, latitude along *x*-axis, longitude along *y*-axis.

**FIGURE 2:**
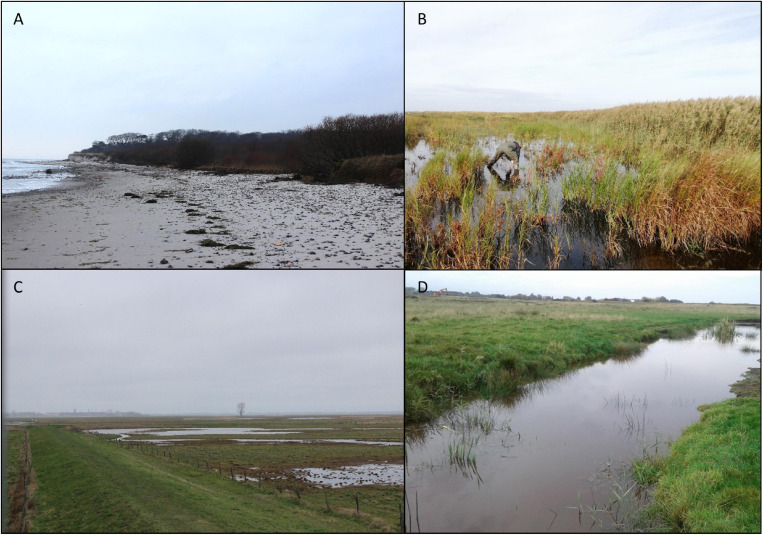
Representative pictures of the four sampling sites **(A)** Hütelmoor beach, **(B)** Hütelmoor peatland, **(C)** Karrendorf peatland, and **(D)** Drammendorf peatland.

Undisturbed sediment cores (diameter: 5 cm; length: 10 cm) taken in May/June 2019 from four study sites (Figure 1) were used for benthic diatom isolation. Two sediment cores originated from the nature reserve “Heiligensee und Hütelmoor” northeast of Rostock, Mecklenburg Pomerania, Germany. The first core was taken on a sandy beach side (54.22550N, 12.17185E) in close vicinity to the Baltic Sea (Figure 2A). Salinity, temperature, and water availability highly fluctuate, as the sampling point gets flooded irregularly with Baltic Sea water. The second core was taken within the coastal peatland (54.21212N, 12.18343E) at a water depth of 20–30 cm (Figure 2B). Water temperature ranged from ∼4°C in winter to ∼26°C in summer (2019), while salinity was relatively stable between 2.5 and 5 S_A_ (2019). The third sampling core was taken from the peatland Karrendorf (Figure 2C), in a small trench at 20–30 cm water depth (54.15796N, 13.38859E). In winter, water temperature ranged around 5°C and salinity at 6–7 S_A_ (2019). There are no data available on highest water temperature in summer for this sampling point as it fell dry. Measurements of a similar sampling point about 100 m west (54.15781N, 13.39241E) indicate water temperature of ∼30°C and salinities of up to 18 S_A_ in summer before falling dry. The fourth core was taken in the peatland Drammendorf (Figure 2D) from a small trench at about 20–30 cm water depth (54.36971N, 13.24384E). Water temperature in these trenches before flooding of this area and at the time of sampling ranged between ∼4°C in winter and ∼30°C in summer, and salinity ranged between 0.5 and 3.5 S_A_.

### Cultures Establishment and Culture Conditions

The upper 1 cm layer of each sediment core served as basis for diatom isolation. 2.3 cm^3^ of fresh sediment were incubated for approximately 2 weeks in Guillard’s f/2 medium (Guillard and Ryther, 1962; Guillard, 1975) enriched with metasilicate (Na_2_SiO_3_ ⋅5 H_2_O; 10 g 100 ml^–1^) to a final concentration of 0.6 mM (further referred to as culture condition). The basis culture medium was Baltic Sea water (∼12 S_A_) enriched with synthetic sea salt (hw Marinemix^®^ professional, hw Wiegandt Aquaristik, Krefeld, Germany) to achieve a final salinity of 15 S_A_. Culture conditions were set to 20°C at 30–50 μmol photons m^–2^ s^–1^ under a 16:8 h light:dark cycle provided by Osram Daylight Lumilux Cool White lamps L36W/840 (Osram, Munich, Germany). After microscopic inspection and ca. 2–4 weeks incubation, single cell isolation was performed under sterile conditions by manual cell picking of the most abundant benthic diatoms and their repeated transfer into fresh cultivation medium until unialgal cultures were established. Isolates were maintained as clonal, but not axenic cultures—DAPI-staining [4’,6-diamidino-2-phenylindole, a blue-fluorescent DNA stain for bacteria (Carl Roth, Karlsruhe, Germany)] typically resulted in low bacteria numbers ranging in maximum of 0.05–1% of the diatom biomass. From each of these sampling sites, one representative isolate was used for the experimental set-ups. These species were later identified as: *Melosira nummuloides* C. Agardh 1824 (Hütelmoor, Beach), *Planothidium* sp. (st. 1) Round and L. Bukhtiyarova, 1996 (Hütelmoor, peatland), *Planothidium* sp. (st. 2) Round and L. Bukhtiyarova, 1996 (Karrendorf) and *Nitzschia filiformis* (W. Smith) Van Heurck 1896 (Drammendorf).

All strains were identified using morphological and molecular approaches. Morphological identification was supported using light microscopic images of living and valve cells, as well as scanning electron microscope (SEM) images. Isolates were treated in 15% H_2_O_2_ to remove most organic contents resulting in the detachment of the epi- and hypotheca of most of the cells. Valves were coated with an ultrathin layer of graphite by high-vacuum evaporation (Leica EM SCD 500; Fa. Leica, Bensheim) for electrical conductivity. Images were taken with a Field Emission Scanning Electron Microscope MERLIN^®^ VP Compact (Fa.Zeiss, Oberkochen) and the SmartSEM^®^ program (version 5.09; Fa. Zeiss, Oberkochen) at varying magnifications of 1500×–15,000×.

Recent literature was used for species identification (Hofmann et al., 2013). Identified species were compared to the data base Algae Base^1^ for recent nomenclature. Molecular analysis followed the approach of Abarca et al. (2014) using the primer Diat-rbcL-R and Diat-rbcL-iF for the *rbc*L gene. PCR mix (20 μl) consisted of 10 μl MyTaq^TM^ mix (Bioline), 6 μl HPLC H_2_O, 1 μl of each primer (20 μM) and 2 μl DNA sample, following the PCR regime of Abarca et al. (2014). Sanger sequencing was conducted by Microsynth Seqlab GmbH, Göttingen, Germany. All sequences were submitted to the National Center for Biotechnology Information (NCBI) under the following accession numbers: *Melosira nummuloides* (MW070612) (strain PTM9a), *Planothidium* sp. (st. 1) (MW070614) (strain PTM25), *Planothidium* sp. (st. 2) (MW070611) (strain PTM7), and *Nitzschia filiformis* (MW070613) (strain PTM10). These cultures are available at the culture collection of the Department Applied Ecology and Phycology, University of Rostock.

### Growth Rates

To determine growth rates of the four benthic diatoms strains in dependence of temperature and salinity, the fluorescence of chlorophyll *a* was used as proxy for biomass. *In vivo* fluorimetry is a non-invasive, simple, and robust method, which can be applied to adhering and filamentous microalgae, and is particularly suitable for benthic diatoms (Karsten et al., 1996; Gustavs et al., 2009). *In vivo* chlorophyll *a* fluorescence measurements as proxy for growth were performed with a self-constructed growth fluorimeter based on the basic electronic unit of a MFMS fluorimeter (Hansatech Instruments, King’s Lynn, United Kingdom) according to the protocol of Karsten et al. (1996). Bright-blue light LEDs emission (Nichia, Nürnberg, Germany) with a peak emission wavelength at 470 nm were selected for excitation of the chlorophyll *a* fluorescence and pulsed with a modulation frequency of 870 Hz. Chlorophyll *a* fluorescence was detected as relative units by an amplified photodiode and was separated from scattered excitation light through a long pass glass filters (RG 665; Schott, Mainz, Germany) and a bright-red gelatin filter (Lee, Brussels, Belgium). *In vivo* chlorophyll *a* fluorescence units correlate very well to cell number, organic carbon and chlorophyll *a* concentration in diatoms as shown by Karsten et al. (1996) and Gustavs et al. (2009).

The cultures were grown in disposable petri dishes with cover lids in a volume of 15 ml culture medium and measured every 24 h for 10 days following the procedure of Gustavs et al. (2009). All cultures were acclimated to the new culturing conditions for 4 days before the experiments. Light was kept constant at 25 μmol photons m^–2^ s^–1^ following a 16:8 light:dark cycle (Osram light sources see above). The cultures were kept in water baths or air-conditioned rooms to ensure constant temperature conditions, all experiments were done in triplicates. Six temperatures (5, 10, 15, 20, 25, and 30°C) were tested at a salinity of 15 S_A_, which corresponded to the standard cultivation conditions (see above). To determine the growth rate dependence on salinity, six salinities were chosen: 1, 5, 10, 15, 27, and 39 S_A_; incubation temperature was kept constant at 20°C. The salinities were adjusted using synthetic sea salt (hw-Marinemix^®^ professional) dissolved in aqua dest. and enriched with f/2 and metasilicate according to standard cultivation conditions (see above). Growth rates were calculated separately for every replicate using the phase, where the fluorescence signal increased exponentially, applying the following equation: N = N_0_^∗^ e^(μ^ ^* dt)^ (N—fluorescence at the measuring day, dt—difference of time in days between measuring day and starting day, μ—growth rate) (Gustavs et al., 2009).

### Light Response Curves (PI-Curves)

Photosynthesis-irradiance (PI)-curves of the four diatom strains were measured according to Prelle et al. (2019). Shortly, 3 ml of thin log phase algal suspension (to avoid self-shading chlorophyll *a* content was kept at 1,270 μg/l on average) of each strain and 30 μl sodium bicarbonate (NaHCO_3_ 2 mM final concentration) were added to four airtight water-tempered (20°C) oxygen electrode chambers (DW1, Hansatech Instruments, King’s Lynn, United Kingdom). At 10 increasing photon flux density levels ranging from 0 to ∼1,500 μmol photons m^–2^ s^–1^ of photosynthetically active radiation (PAR), oxygen concentration was measured using a non-invasive oxygen dipping probe (DP sensors PreSens Precision Sensing GmbH, Regensburg, Germany). Measurements consisted of a 30 min respiration phase, followed by a 10 min photosynthesis phase for each light level. The first and last minute of each measurement were excluded from the calculation. After the last measurement, chlorophyll *a* was extracted from the 3 ml algal suspension using 96% ethanol and quantified spectrophotometrically at 665 nm (Shimadzu UV-2401 PC, Kyoto, Japan) (HELCOM, 2015).

The mathematical photosynthesis model of Walsby (1997) was used for fitting and calculation of maximum rates of net primary production (NPP_max_), respiration (R), light utilization coefficient (α), photoinhibition coefficient (β), light saturation point (I_k_) and the light compensation point (I_c_).

### Temperature-Dependent Photosynthesis and Respiration

Following the methodological approach of Karsten et al. (2010), the photosynthetic and respiratory response of each strain at temperatures between 5 and 40°C was measured using the same oxygen optode system as for the PI-curves. After 20 min incubation in the dark, the respiratory oxygen consumption (10 min in the dark), followed by the photosynthetic oxygen production (10 min under light saturated conditions at 320 ± 45 μmol photons m^–2^ s^–1^ PAR) were determined. Measurements were normalized to the total chlorophyll *a* concentration (HELCOM, 2015). Photosynthesis to respiration ratios (P:R) were calculated, excluding positive respiration and negative photosynthesis measurements.

### Cell Biology

To investigate the effects of salinity and temperature on the formation of diatom frustules and the storage of lipids, the four diatom strains were incubated under different salinity and temperature conditions. Salinity effects were investigated in cultivation media of 1, 15, and 30 S_A_ at 20°C, temperature effects at 7, 17, and 22°C at 15 S_A_ (see section “*Growth Rates* for More Details on Cultivation Conditions”).

Cultures were pre-incubated for 4 days in disposable petri dishes with cover lids (20 ml) at the respective experimental growth conditions to acclimate and to attain log-phase. After pre-incubation, 5 ml of each culture were added into 6-well cell culture plates (Greiner BioOne) to the respective fresh medium (final volume 15 ml) and were incubated for another 7 days.

Setting the samples for the investigation of the frustule formation toward temperature and salinity, PDMPO ([2-(4-pyridyl)-5-((4-(2-dimethylaminoethylaminocarbamoyl) methoxy)phenyl)oxazole]) was used as stain for visualization according to Shimizu et al. (2001). A 100 μmol l^–1^ PDMPO [LysoSensor(^TM^) Yellow/Blue DND-160” (Invitrogen, Carlsbad, United States)] working solution was prepared with DMSO (Dimethylsulfoxid) (Calbiochem, San Diego, United States) and added to each of the 6-well cell culture plate with a final concentration of 1 μmol l^–1^. The PDMPO was incorporated into newly formed valves and visualized with an epi-fluorescence microscope. To quantify new valve formation, fixed samples were observed at 400× total magnification using epi-fluorescence microscopes (BX 51 and IX 70, Olympus, Hamburg, Germany) under UV excitation (U-MWU, excitation: 330–385 nm, emission: > 420 nm, Olympus, Hamburg, Germany). In each sample, 400 cells were examined and categorized into: (1) at least one valve stained, (2) no valve stained.

On days 3 and 7, 1 ml culture was taken from each well and immediately fixed with glutardialdehyde (2% final concentration) and stored in the dark until further treatment.

The effects of temperature and salinity on lipid formation and lipid volume of the four diatom isolates were investigated using nile red lipid staining according to Greenspan et al. (1985). To each 1 ml sample, 1 μl of nile red (Carl Roth, Karlsruhe, Germany) was added. After 10 min of dark incubation, each sample was evaluated at 400-fold magnification using an epi-fluorescence microscope (BX-51, Olympus, Hamburg, Germany) under blue excitation (U-MWB, Olympus, Hamburg, Germany). Micrographs of 50 cells [*Planothidium* sp. (st. 1) and *Planothidium* sp. (st. 2)] or 20 cells (*M. nummuloides* and *N. filiformis*) per sample were taken using CellSens Standard imaging software (Olympus, Hamburg, Germany). The diameter of each lipid droplet was measured using ImageJ (Open Source by National Institutes of Health) and volume of the respective shape (oval, conical and spherical shapes) was calculated.

### Statistical Analysis

All calculations and figures were made using Microsoft Office Excel (2016)–partly using the solver function by minimizing the sum of normalized squared deviations–and R (Version: 4.0.2). Statistical analysis was performed using R. Growth rates as function of temperature and temperature-dependent photosynthesis and respiration were fitted using the model of Yan and Hunt (1999), which proved to be best suitable for simple biological processes (Adams et al., 2017). Growth rates as function of salinity were not fitted due to almost no response. Confidence intervals were calculated using the library nlstools in R and are provided in Supplementary Table 1. Significance levels were calculated using one-way ANOVA followed by a *post hoc* Tukey’s honest significant differences test (critical *p* < 0.05).

## Results

### Species Identification

**FIGURE 3:**
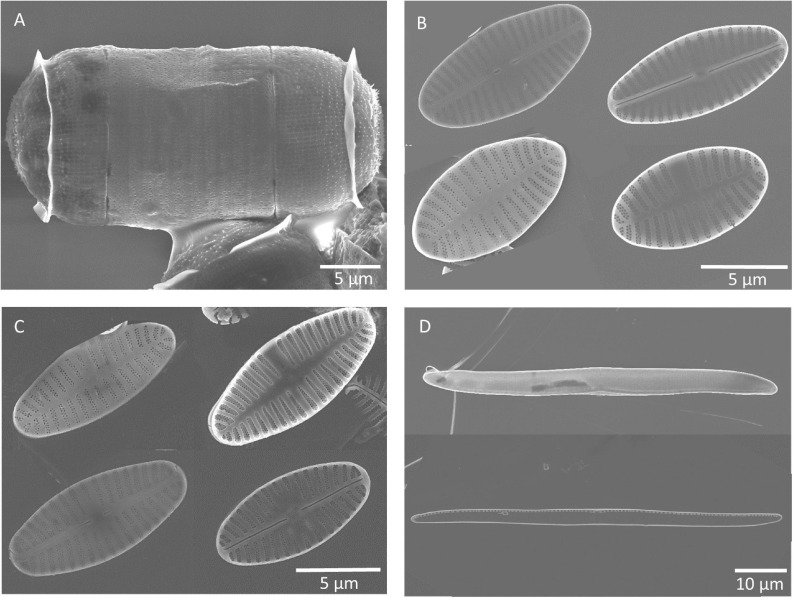
Scanning electron microscopy images (by Kana Kuriyama) of frustules of the four benthic diatom strains investigated, **(A)** *Melosira nummuloides*, **(B)** *Planothidium* sp. (st.1) (monoraphid—displaying raphid and araphid valve (in- and outside), **(C)** *Planothidium* sp. (sp. 2) (monoraphid—displaying raphid and araphid valve (in- and outside), **(D)** *Nitzschia filiformis* (in- and outside).

The diatom strains were morphologically identified based on SEM images as *Melosira nummuloides* due to chain formation, as *Nitzschia filiformis* due to its characteristic genus-specific fibulae, and two *Planothidium* sp. (Figures 3A–D). The latter could only be identified to genus level due to unclear morphological traits for species discrimination.

Molecular analysis of the species *M. nummuloides* using NCBI BLASTn (Altschul et al., 1990) coincided to 99% with the *rbc*L gene of *Melosira nummuloides* C67 (FJ002129). Similarly, *N. filiformis* coincided to 99% with the *rbc*L gene of *Nitzschia filiformis* UTEX FD267 (HQ912453) (Theriot et al., 2010). *Planothidium* sp. (st. 1) and *Planothidium* sp. (st. 2) had a 100% overlap of the *rbc*L gene sequences to each other, indicating that both strains belong to the same species. The most similar sequences were *Halamphora americana* (95%, MK045450) and *Planothidium suncheomanense* (94%, KY650831). In combination with the morphological data, we identified the two strains only to the genus level *Planothidium*.

### Growth Rates

**FIGURE 4:**
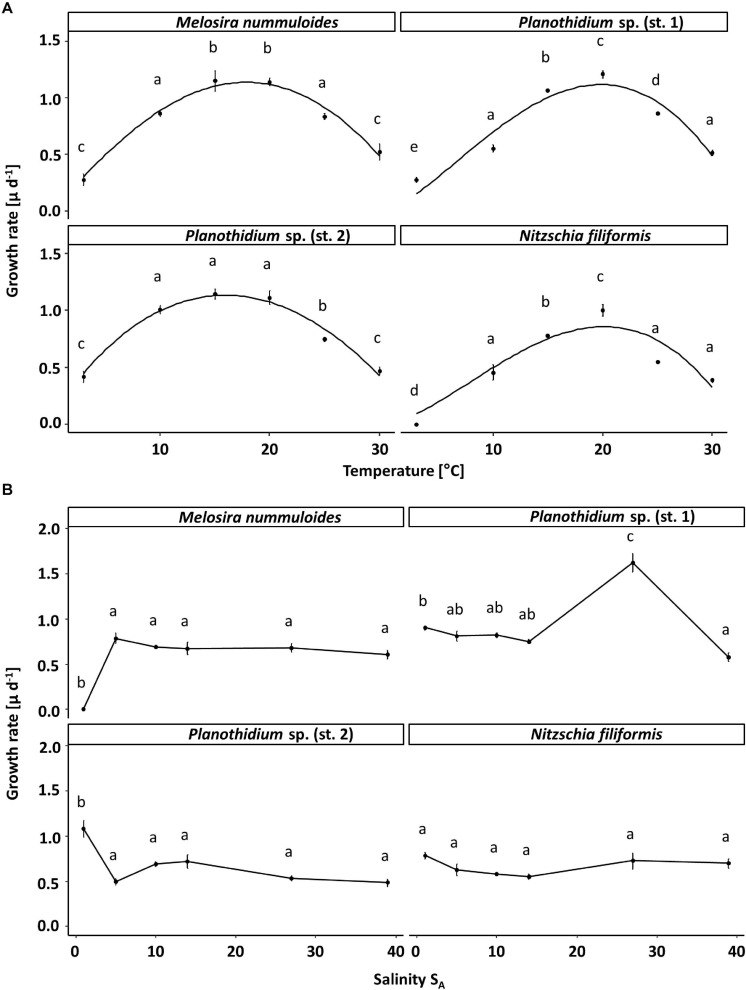
Growth rates (μ d^–1^) in relation to **(A)** temperature and **(B)** salinity of the respective four benthic diatom strains *Melosira nummuloides, Planothidium* sp. (st.1), *Planothidium* sp. (sp. 2) and *Nitzschia filiformis*. Data represent mean values ± SD (*n* = 3). Different lowercase letters represent significant levels among all means as calculated per temperature or salinity by a one-way ANOVA (Tukey’s test, *p* < 0.05). Growth rates in dependence of temperature were fitted using the model of Yan and Hunt (1999).

The temperature effect on the growth rates varied between the four benthic diatom strains (Figure 4). *Nitzschia filiformis* did not grow at 5°C, whereas the other strains showed growth rates up to 0.4 μ d^–1^ at the lowest tested temperature. All strains were able to grow at the highest tested temperature of 30°C, also with growth rates at around 0.5 μ d^–1^. The optimum temperature of *N. filiformis* and *Planothidium* sp. (st. 1) was determined at 20°C with growth rates of 0.86 and 1.11 μ d^–1^, respectively. *Melosira nummuloides* grew best at 15 and 20°C with maximum growth rate of 1.13 μ d^–1^, *Planothidium* sp. (st. 2) between 10 and 20°C with maximum growth rate of 1.14 μ d^–1^ (Figure 4A). All strains exhibited a broad temperature range for optimal growth, where still 80% of the maximum growth rate is reached. *Nitzschia filiformis* and *Planothidium* sp. (st. 1) showed optimum growth between 13 and 26°C, *Planothidium* sp. (st. 1) between 8.4 and 24°C, *M. nummuloides* between 10 and 25°C.

The four tested diatom strains tolerated a broad salinity spectrum. *Planothidium* sp. (st. 2) had the highest growth with a rate of 1.1 μ d^–1^ at 1 S_A_. No significant differences between the other tested salinities were observed. The growth rate of *N. filiformis* was similar at the salinities between 1 and 39 S_A_. *Melosira nummuloides* did not grow at 1 S_A_ but grew well with a rate of 0.8 μ d^–1^ at all other tested salinities between 5 and 39 S_A_. *Planothidium* sp. (st. 1) showed a clear optimum salinity of 27 S_A_ for growth (Figure 4B).

### Light-Dependent Photosynthesis

**FIGURE 5:**
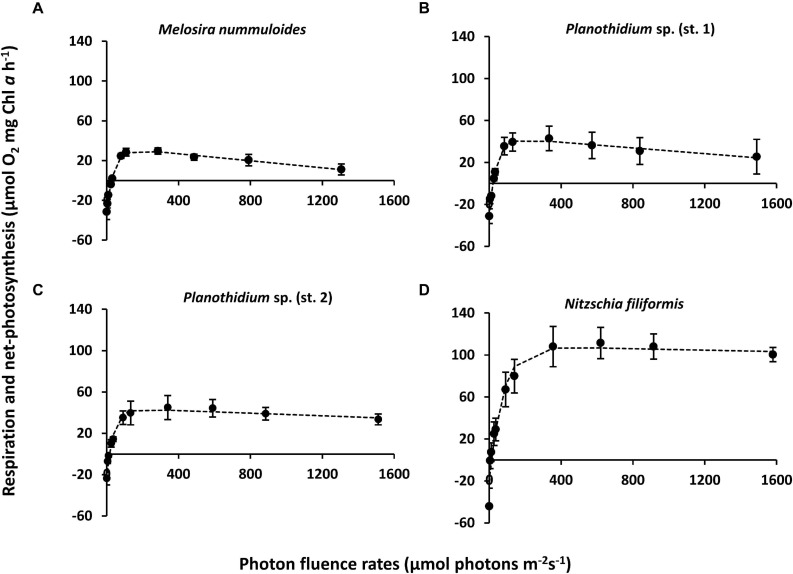
Photosynthesis and respiration rates (μmol O_2_ mg^–1^ chl *a* h^–1^) as function of increasing photon flux density (μmol photons m^–2^s^–1^) of four benthic diatom strains kept at 20°C in f/2 Baltic Sea medium, 15S_A_ and measured by oxygen optodes. Data represent mean values ± SD (*n* = 3, except *Planothidium* sp. 1 *n* = 4). **(A)** *Melosira nummuloides*, **(B)** *Planothidium* sp. (st. 1), **(C)** *Planothidium* sp. (st. 2), **(D)** *Nitzschia filiformis.* Data points were fitted by the model of Walsby (1997).

Photosynthesis and respiration of the four benthic diatom strains resulted in strain-specific responses as function of increasing photon fluence rates up to ∼ 1,500 μmol photons m^–2^ s^–1^ (Figure 5). Due to low numbers of diatom-associated bacteria (<0.05–1% of diatom biomass), oxygen signals can be ascribed to the diatom response during experimentation.

Characteristic PI-curve parameters were calculated using the photosynthetic model of Walsby (1997) (Table 1). *Planothidium* sp. (st. 2) exhibited the lowest dark respiration rate (−23.75 μmol O_2_ mg^–1^ chl *a* h^–1^) and *N. filiformis* the highest (−48.64 μmol O_2_ mg^–1^ chl *a* h^–1^), with the remaining two isolates between these values. The maximum photosynthetic rates (NPP_max_) of *Planothidium* sp. (st. 2) and *Planothidium* sp. (st. 1) were in a similar range, while the NPP_max_ of *M. nummuloides* was only 50% of these rates and for *N. filiformis* 2.5 times higher. A similar pattern was found for the light saturation point (I_k_) with *Planothidium* sp. (st. 2) exhibiting the lowest value at 36.5 μmol photons m^–2^ s^–1^ and *N. filiformis* the highest value at 56.1 μmol photons m^–2^ s^–1^. The highest light utilization coefficient (α) was calculated for *N. filiformis* with 2.77 μmol O_2_ mg^–1^ chl *a* h^–1^ (μmol photons m^–2^ s^–1^) ^–1^ The remaining isolates had slightly lower α, ranging from 1.68 to 2.21 μmol O_2_ mg^–1^ chl *a* h^–1^ (μmol photons m^–2^ s^–1^) ^–1^. Compared to *Planothidium* sp. (st. 2) and *N. filiformis* light compensation points (I_c_) were slightly higher in *M. nummuloides* and *Planothidium* sp. (st. 1), but all ranged between 14.0 and 26.1 μmol photons m^–2^ s^–1^. Using the Walsby fit (1997), photoinhibition was detected in *M. nummuloides* and both *Planothidium* sp. cultures. NPP_max_ to respiration ratios of the four isolates ranged from 0.97 to 5.07 (Figure 5 and Table 1).

**TABLE 1:** Parameter of respective *PI*-curves (Figure 5) of four benthic diatom strains [*n* = 3, except *Planothidium* sp. (st. 2) *n* = 4] kept at 20°C in a f/2 Baltic Sea medium, 15 S_A_.

Isolates	NPP_max_ (μmol O_2_ mg^–1^ chl *a* h^–1^)	Respiration (μmol O_2_ mg^–1^ chl *a* h^–1^)	α (μmol O_2_ mg^–1^ chl *a* h^–1^) (μmol photons m^–2^ s^–1^) ^–1^	β (μmol O_2_ mg^–1^ chl *a* h^–1^) (μmol photons m^–2^ s^–1^) ^–1^	I_k_ (μmol photons m^–2^ s^–1^)	I_c_ (μmol photons m^–2^ s^–1^)	NPP_max_: Respiration
*Melosira nummuloides*	30.42 ± 0.22 a	−31.42 ± 1.89 a	1.68 ± 0.72 a	−0.02 ± 0.00 a	36.66 ± 15.23 a	26.09 ± 13.44 a	0.97 ± 0.03 a
*Planothidium* sp. (st. 1)	46.09 ± 8.50 a	−31.49 ± 7.94 a	1.93 ± 0.54 a	−0.01 ± 0.01 a	40.24 ± 6.37 ab	20.95 ± 4.46 a	1.46 ± 0.57 a
*Planothidium* sp. (st. 2)	42.89 ± 10.79 a	−23.75 ± 5.96 a	1.83 ± 0.48 a	−0.01 ± 0.01 a	36.48 ± 2.91 ab	16.10 ± 1.45 a	1.81 ± 0.12 ab
*Nitzschia filiformis*	106.94 ± 17.24 b	−48.64 ± 12.89 a	1.76 ± 0.75 a	0.00 ± 0.02 a	72.60 ± 17.17 b	14.06 ± 8.21 a	5.07 ± 0.49 b

*Different lowercase letters represent significant levels among all means as calculated by a one-way ANOVA (Tukey’s test, p < 0.05). NPP_max_ represents the maximal oxygen production rate, alpha the initial slope of production in the light limited range, beta the terminal slope of production in extensive light range (photoinhibition), I_k_ the light saturation point, I_c_ the light compensation point.*

### Temperature-Dependent Photosynthesis and Respiration

**FIGURE 6:**
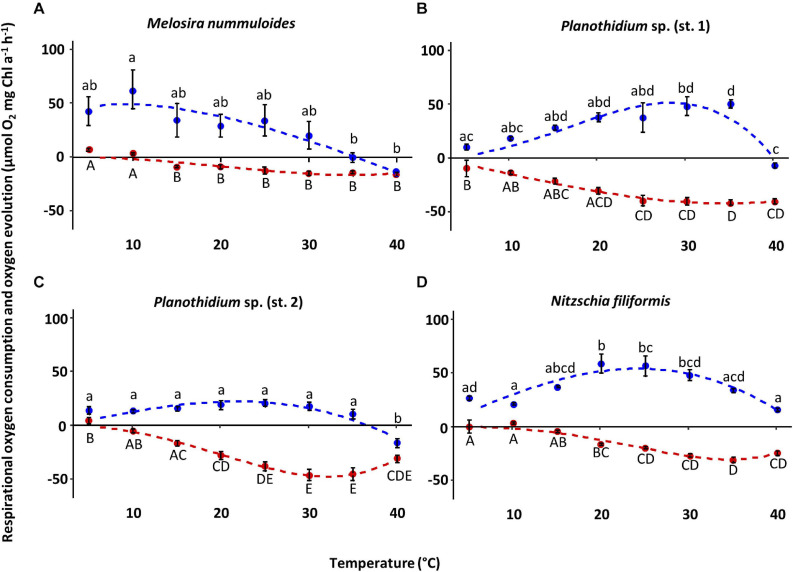
Photosynthetic (blue) oxygen production at 320 ± 45 μmol photons m^–2^s^–1^ and respiratory (red) oxygen consumption in darkness of four benthic diatom strains as a function of increasing temperature. The measured data were fitted by the model of Yan and Hunt (1999) (photosynthesis: blue dashed line; respiration: red dashed line). All cultures were kept in f/2 Baltic Sea medium, 15S_A_. Data represent mean values ± SD (*n* = 3, except *Planothidium* sp. (st. 2) *n* = 4). Different lowercase (photosynthesis) and capital letters (respiration) indicate significant means (*p* < 0.05; one-way ANOVA with *post hoc* Tukey’s test). **(A)** *Melosira nummuloides*, **(B)** *Planothidium* sp. (st. 1), **(C)** *Planothidium* sp. (st. 2), **(D)** *Nitzschia filiformis.*

The four diatom strains exhibited different photosynthetic and respiratory responses to increasing temperatures between 5 and 40°C (Figure 6 and Table 2). The net photosynthetic oxygen production and respiratory oxygen consumption generally increased with rising temperatures up to a strain-specific maximum. With further increasing temperature a decrease in photosynthetic oxygen production and respiratory oxygen consumption was observed. Highest respiration rates were measured between 30 and 40°C among all species while photosynthetic optima varied strongly from 10 to 35°C (Figure 6). The overall oxygen production of *Planothidium* sp. (st. 2) showed only weak dependence to temperature and was lowest compared to the remaining three strains with 20.0 μmol O_2_ mg^–1^ chl *a* h^–1^ at 25°C (Figure 6). In contrast, *N. filiformis* exhibited the overall highest photosynthetic maximum amongst the four strains with 59.1 μmol O_2_ mg^–1^ chl *a* h^–1^ at 20°C. In contrast to the other three isolates *M. nummuloides* did not follow the trend of an increasing oxygen production with increasing temperatures by reaching its maximum production of 62.8 μmol O_2_ mg^–1^ chl *a* h^–1^ already at 10°C. Between 10 and 25°C photosynthesis decreased almost linearly (Figure 6). Modeled data of all four strains showed optimal photosynthesis (defined as 80% percentile) over a species-specific temperature span between 13 and 18°C, while reduced photosynthesis (defined as 20% percentile) was performed over a span between 29 and 37°C (Table 2).

**TABLE 2:** Results of model calculation for temperature-dependent growth rate, photosynthetic rate, and respirational rate following the model of Yan and Hunt (1999).

			*Melosira nummuloides*	*Planothidium* sp. (st. 1)	*Planothidium* sp. (st. 2)	*Nitzschia filiformis*
Growth	Maximal growth rate		1.1	1.1	1.1	0.9
	Optimum temperature		17.9	19.8	16.0	20.1
	Maximum temperature		33.8	33.1	33.8	32.4
	Residual sum-square		0.1	0.2	0.1	0.3
	Temperature range for	Optimal growth (80% growth rate)	10.4–25.1	12.9–26	8.4–23.8	13.5–26
		Growth (20% growth rate)	2.3–32.2	4.0–31.8	4.6–31.2	4.6–31.2
Photosynthesis	Maximal photosynthetic rate		49.1	51.6	22.4	54.2
	Optimum temperature		9.1	28.2	21.8	24.2
	Maximum temperature		35.3	39.9	36.6	42.7
	Residual sum-square		9,028	3,292	1,429	2,165
	Temperature range for	Optimal photosynthesis (80% photosynthetic rate)	2.5–19.1	21–33.9	14.1–28.8	14.9–32.7
		Photosynthesis (20% photosynthetic rate)	0.1–31.9	9.8–38.8	4.3–35.3	4–40.9
Respiration	Maximal respirational rate		−17.0	−42.2	−47.8	−30.6
	Optimum temperature		34.8	34.6	32.2	34.6
	Maximum temperature		49.1	59.4	44.5	45.4
	Residual sum-square		502.5	861.8	1,389	444.8
	Temperature range for	Optimal respiration (80% respirational rate)	26.2–41.7	21.9–46.1	24.6–38.2	27.6–39.9
		Respiration (20% respirational rate)	12.4–47.8	6.3–56.9	12–43.3	15.2–44.4

Overall, the respisratory oxygen consumption of the four strains increased with beginning to low temperature, but decreased after reaching the strain-specific optimum, which was slightly higher than the photosynthetic optimum (Figure 6 and Table 2). In *Planothidium* sp. (st. 2) and *M. nummuloides* respiratory oxygen consumption at 5°C was not detectable, and the same was true at 10°C for *M. nummuloides* and *N. filiformis* (Figure 6). Maximum respiratory oxygen consumption was measured with −46.9 μmol O_2_ mg^–1^ chl *a* h^–1^ at 30°C for *Planothidium* sp. (st. 2), with −30.3 μmol O_2_ mg^–1^ chl *a* h^–1^ at 35°C for *N. filiformis*, with −42.0 μmol O_2_ mg^–1^ chl *a* h^–1^ at 35°C and with −15.97 μmol O_2_ mg^–1^ chl *a* h^–1^ at 40°C for *M. nummuloides* (Figure 6). The optimum respiration (80% of maximum rate) span between 22 and 40°C, while 20% of maximum respiration rate was reached at 12°C or even lower temperatures for *Planothidium* sp. st.1 (Table 2).

Calculations of P:R ratios exhibited significant differences between the strains at different temperature levels (Table 2). At 15°C, P:R ratio of *N. filiformis* was significantly higher with a value of 9.9 compared to the other temperatures. P:R ratios were otherwise rather similar (0.03–5.4) across all temperatures and strains (Table 3).

**TABLE 3:** P:R ratio of respective temperature dependent photosynthesis and respiration curves (Figure 6) of four benthic diatom strains [*n* = 3, except *Planothidium* sp. (st. 2) *n* = 4 and *M. nummuloides n* = 2] kept at 20°C in a f/2 Baltic Sea medium, 15 S_A_.

Isolates	5°C	10°C	15°C	20°C	25°C	30°C	35°C	40°C
*Melosira nummuloides*	–	–	3.55 ± 2.64 ab	3.52 ± 3.17 a	2.47 ± 1.42 ab	1.21 ± 1.26 a	–	–
*Planothidium* sp. (st. 1)	–	1.39 ± 0.36 a	1.36 ± 0.46 a	1.29 ± 0.43 a	1.06 ± 0.82 ab	1.17 ± 0.22 a	1.20 ± 0.11 a	–
*Planothidium* sp. (st. 2)	–	5.16 ± 6.58 a	1.04 ± 0.43 a	0.73 ± 0.36 a	0.54 ± 0.18 a	0.41 ± 0.21 a	0.25 ± 0.16 b	–
*Nitzschia filiformis*	–	–	9.92 ± 4.67 b	3.61 ± 0.76 a	2.80 ± 0.67 b	1.78 ± 0.37 a	1.14 ± 0.17 a	0.67 ± 0.03

*Different lowercase letters represent significant levels among all means as calculated per temperature by a one-way ANOVA (Tukey’s test, p < 0.05); positive respiration and negative photosynthesis measurements were excluded from ratio calculations.*

### Effects of Temperature and Salinity on Diatom Frustule Formation

**FIGURE 7:**
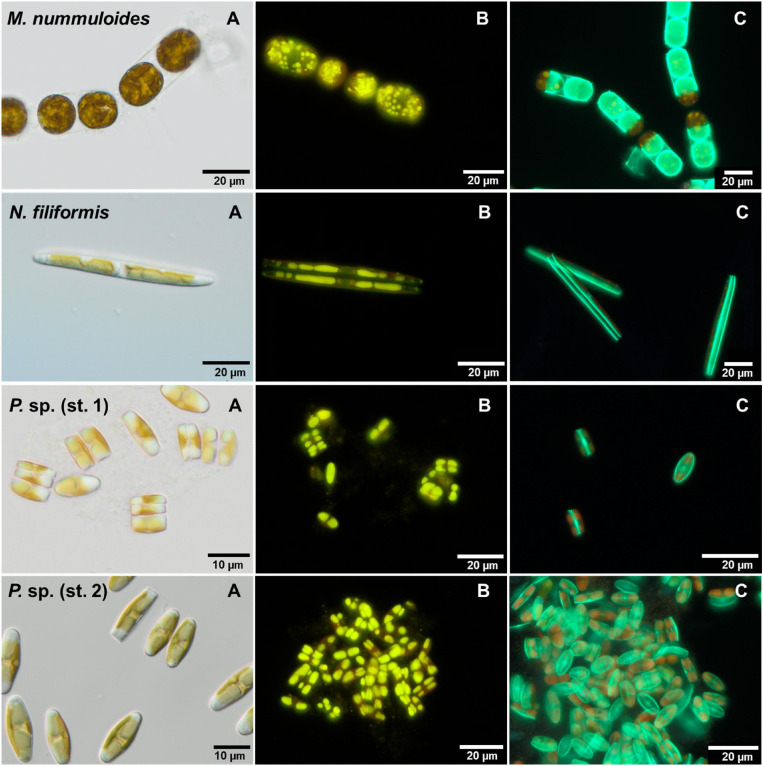
Micrographs of the four benthic diatom strains *Melosira nummuloides*, *Planothidium* sp. (st. 1), *Planothidium* sp. (st. 2) and *Nitzschia filiformis* with **(A)** differential interference contrast, **(B)** stained with nile red and **(C)** 2-(4-pyridyl)-5-((4-(2-dimethylaminoethylaminocarbamoyl)methoxy)phenyl)oxazole stain.

**FIGURE 8:**
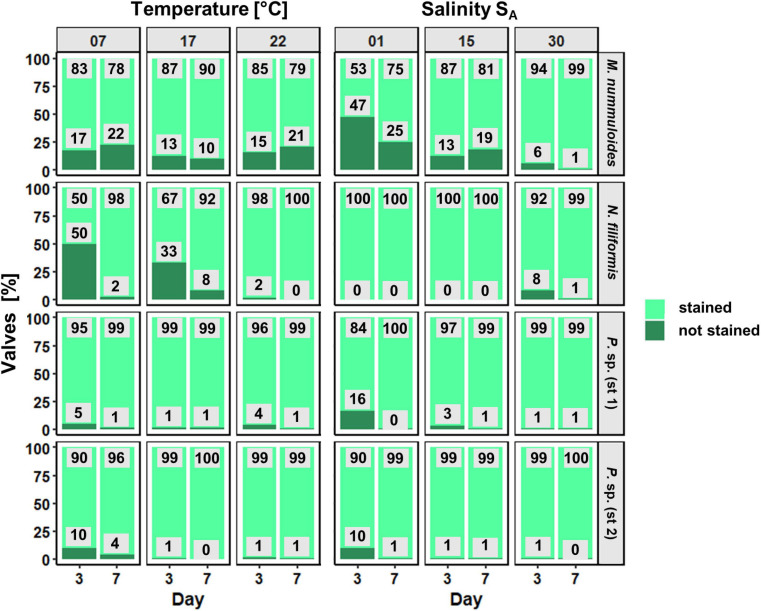
Proportion (in %) of newly formed valves as function of temperature and salinity of four benthic diatom strains *Melosira nummuloides*, *Planothidium* sp. (st. 1), *Planothidium* sp. (st. 2), and *Nitzschia filiformis* for sampling days 3 and 7.

For each isolate, the proportion of PDMPO-stained (new) and unstained diatom valves was counted on 2 days (Figure 7). Depending on the respective growth rate derived from the chlorophyll *a* fluorescence, 1–22% of unstained cells were expected. Overall, the proportion of the newly built valves was at least 50%, even higher in most cases, at both sampling days as the exponential growth mostly occurred until day 5. Furthermore, the overall percentage of newly built valves increased slightly from day 3 to day 7, for both temperature and salinity (Figure 8). *Planothidium* sp. (st. 1) and *Planothidium* sp. (st. 2) (Figure 8) exhibited no major differences in the formation of new valves concerning both sampling days as well as varying temperatures. For *M. nummuloides*, a higher proportion of unstained valves was found at temperatures of 7 and 22°C from days 3 to 7 and a decrease was found for 17°C. At day 3, *N. filiformis* exhibited the overall lowest percentage of unstained valves at 7 and 17°C. At day 7 percentage of newly formed valves strongly increased (Figure 8).

Different salinities as well as the sampling day had almost no effect on the formation of new valves for *N. filiformis, Planothidium* sp. (st. 1) and *Planothidium* sp. (st. 2) (Figure 8). *Melosira nummuloides* exhibited the overall lowest amount of newly formed valves at 1 S_A_ and 15 S_A_, while most new valves were produced at 30 S_A_. Further, an increase of unstained valves from days 3 to 7 was observed for 15 S_A_ (Figure 8).

The PDMPO-staining approach confirmed the ecophysiological data well.

### Effects of Temperature and Salinity on Diatom Lipid Droplets

**FIGURE 9:**
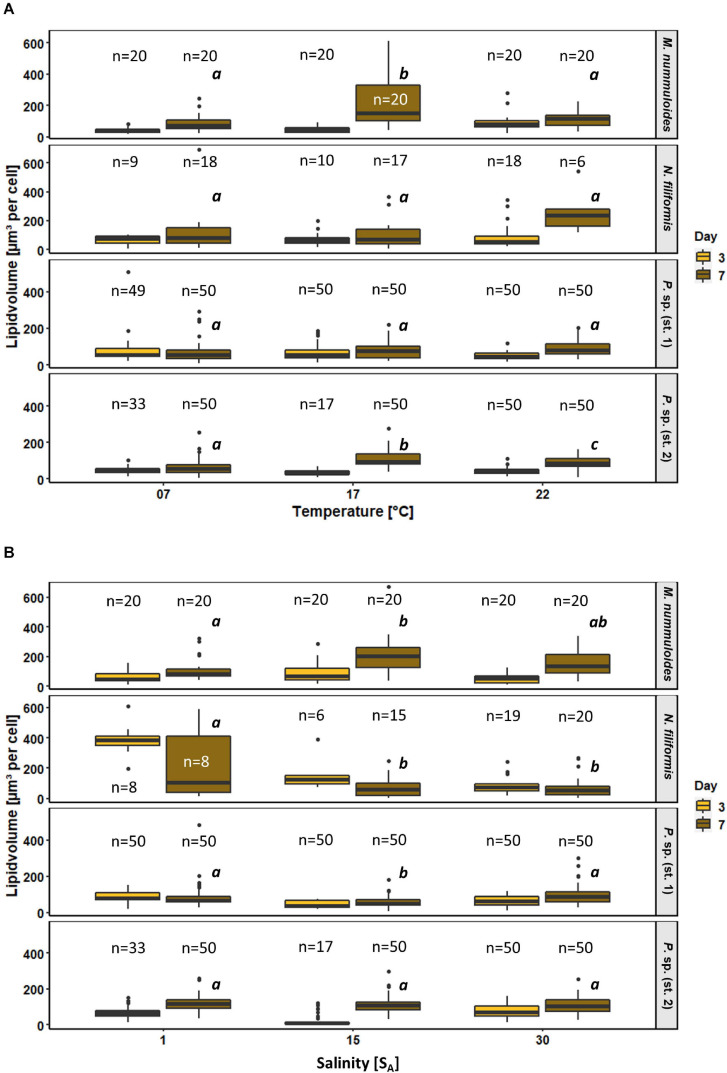
Box plots of lipid volume as function of **(A)** temperature and **(B)** salinity of four benthic diatom strains *Melosira nummuloides*, *Planothidium* sp. (st. 1), *Planothidium* sp. (st. 2), and *Nitzschia filiformis* for sampling days 3 and 7. n indicates the number of observed cells. Letters in italic indicate significant difference between median values of days 3 to 7 of the treatments.

The effects of temperature and salinity on the storage of lipids of the four diatom strains were determined calculating the lipid volume per cell on two separate days (Figure 9). *Melosira nummuloides* and *N. filiformis* stored their lipid droplets in several different shapes within considerably larger cells, while *Planothidium* sp. (st. 1) and *Planothidium* sp. (st. 2) exhibited two marginal lipid droplets within smaller cells (Figure 7). The effects of the different treatments within each strain were compared and tested for significance at day 7.

All strains exhibited rather uniform lipid volumes at day 3 during exponential growth for all temperature treatments. During the stationary phase at day 7, increases in lipid volume median over time were found for all tested strains at 17 and 22°C, ranging from 35 to 340%. At 7°C smaller or no changes occurred. Temperature effects were significant at day 7 for *M. nummuloides* and *Planothidium* sp. (st. 2) at 17°C, with the latter also being significantly changed at 22°C (Figure 9). *Planothidium* sp. (st. 1) did not show major lipid volume changes at the different temperatures. *Nitzschia filiformis* exhibited high variability, which led to mostly no statistical significance.

Salinity had contrasting and significant effects on the lipid volume of *M. nummuloides*, *N. filiformis* and *Planothidium* sp. (st. 1) (Figure 9). The lipid volume increased between days 3 and 7 in *M. nummuloides* and *Planothidium* sp. (st. 2) at all tested salinities from 8 to 204 μm^3^ per cell. *Planothidium* sp. (st. 1) showed minor differences between day 3 and day 7 with ambiguous results: a slight 20% decrease in lipid volume at 1 S_A_ and slight 40% increases at 15 and 30 S_A_. In *N. filiformis* the lipid volume decreased for all three salinities over time. Significant changes in lipid volume after 7 days of treatment were found in all strains except *Planothidium* sp. (st. 2) but with different patterns as *N. filiformis* had the highest median lipid volume (387 μm^3^ per cell) at the lowest salinity while *M. nummuloides* had its highest median (204 μm^3^ per cell) at 15 S_A_.

**FIGURE 10:**
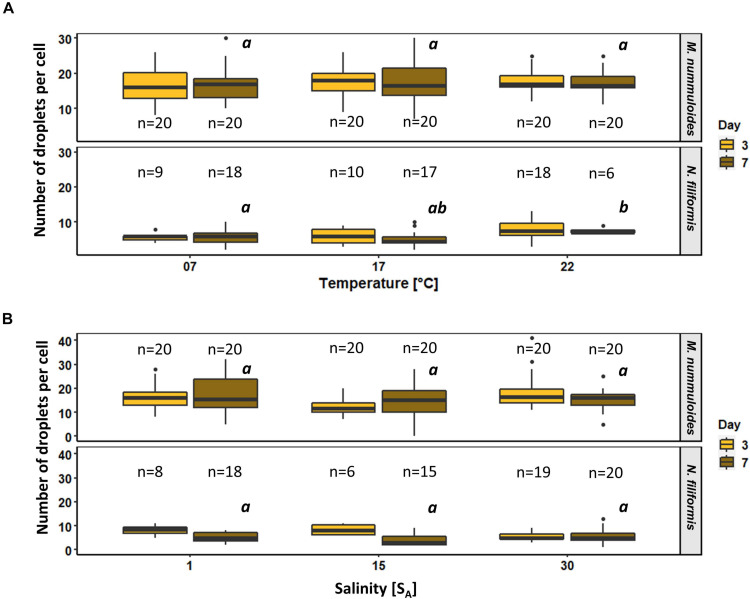
Box plots of number of lipid droplets per cell as function of **(A)** temperature and **(B)** salinity of two diatom strains *Melosira nummuloides* and *Nitzschia filiformis* for sampling days 3 and 7. n indicates the number of observed cells. Letters in italic indicate significant difference between median values of days 3 to 7 of the treatments.

To determine the effects of temperature and salinity on the amount of lipid droplets per cell, lipid droplets were counted on both sampling days. *Planothidium* sp. always holds two lipid droplets. Therefore, only *M. nummuloides* and *N. filiformis* were included for in depth examination (Figure 10). The average amount of lipid droplets per cell for *M. nummuloides* was 16 and exhibited only slight changes from days 3 to 7 and no significant differences for all temperature and salinity treatments at day 7. *Nitzschia filiformis* showed relatively constant numbers of lipid droplets (3–8) between days 3 and 7 for the different temperatures but a 30% decrease for the lower two salinities, whereas at 30 S_A_ the number of droplets was nearly equal. The comparison of the treatment effects of temperature and salinity, respectively, at day 7 revealed changes depending on the temperature but no significant effects of salinity.

## Discussion

The four investigated benthic diatom strains from the coastal Baltic Sea exhibited broad ecophysiological tolerances to the abiotic parameters light, temperature, and salinity, which in this study mimicked the annual fluctuations and extreme events at the study sites. The data presented support our assumption that these isolates are not only very well adapted to these stressors but are also able to withstand future abiotic changes that originate from expanding sea-land transition zones.

### Growth

Salinity at the respective four study sites ranged between 9.2 and 13.6 S_A_ for the Baltic Sea and 1.2–8.9 S_A_ for the peatlands in 2019. In general, the four investigated benthic diatom strains exhibited growth rates ranging from 0.5 to 1.6 μ d^–1^ at salinities from 1 S_A_ up to 39 S_A_ (except for *M. nummuloides*), which far exceeded the range of salinities occurring in their natural habitat by far. This is similar to the study of Woelfel et al. (2014), in which three benthic diatom species from a more westerly site of the Baltic Sea exhibited growth rates between 0.3 and 1.5 μ d^–1^ at salinities ranging from 1 to 50 S_A_. In accordance with our assumption, benthic diatom strains from the coastal peatlands are indeed euryhaline, growing well along salinity gradients between 1 and 39 S_A_, and are therefore well adapted to recurring storm floods with saltwater intrusion into the freshwater habitat.

Although *M. nummuloides* is still considered euryhaline, it was not able to grow at 1 S_A_. *Melosira nummuloides* is known as a marine species (Crawford, 1973) leading to the assumption that it preferentially grows under higher salinities. This species was found on the sandy beach site of the Baltic Sea along the newly developed transition zone between the peatland Hütelmoor and the Baltic Sea after the latest storm surges, which gets flooded irregularly. This broad salinity tolerance of our *M. nummuloides* isolate in conjunction with intolerance of very low salinities confirms its marine characteristics.

*Planothidium* sp. (st. 1) exhibited its optimum growth rate at marine conditions, while salinity at the brackish location of origin normally only ranged between 2.5 and 5 S_A_. Most of the *Planothidium* species are considered as freshwater species, however, there are a few known marine taxa (Algae Base). Therefore, the occurrence of *Planothidium* strains in the peatlands in combination with their salinity optimum may lead to the assumption that *Planothidium* sp. (st. 1) was swept into the peatland during the last saltwater inflow event in January 2019. This is in agreement with a recently submitted study on microphytobenthic primary production at an exposed sandy beach next to the Hütelmoor (Kuriyama et al., 2021), in which the authors report *Planothidium delicatulum* as most the abundant species (25% of the community) attached to sand grains. Even though salinity of the coastal German Baltic Sea in Mecklenburg-Pomerania rarely exceeds 14 S_A_ (Lippert et al., 2017) and therefore is not considered a marine habitat, growth response of other marine species (Algae Base) found in the Baltic Sea such as *Navicula perminuta* (Woelfel et al., 2014), indicate that euryhaline marine species are also able to live in brackish environments.

In contrast to *Planothidium* sp. (st. 1), *Planothidium* sp. (st. 2), which was isolated from the peatland in Karrendorf with salinity ranging between 6 and 8 S_A_, exhibited its maximum growth rate at 1 S_A_. As there were no genetic differences in the *rbcL* gene between both *Planothidium* strains, they can be considered as identical species. The conspicuous differences in the growth vs. salinity patterns, however, can be explained as development into two salinity ecotypes. The study site in Karrendorf has been regularly flooded for almost three decades allowing marine species that have been swept into this peatland to adapt to brackish/freshwater conditions. The concept of ecotypic differentiation is widely accepted in terms of physiological diversity and plasticity in seaweeds along the horizontal salinity gradient of the Baltic Sea (Nygard and Dring, 2008; and references therein), but also for microalgae such as *Skeletonema marinoi* from other regions (Saravanan and Godhe, 2010; and references therein). Ecotypes are variants of a species that are locally adapted to particular environmental conditions.

The brackish species *N. filiformis* (Algae Base) did not show significant differences in growth between 1 and 39 S_A_. In contrast, Trobajo et al. (2011) found that an isolate of *N. filiformis* collected in the Ebro Estuary (Spain) was negatively influenced by salinities above 16 and 22 S_A_ with a slight decrease in growth, although precise growth rates were not provided. In addition, Trobajo et al. (2011) identified their isolate as *N. filiformis* var. *conferta* which probably is genetically different to our Baltic Sea strain.

The cell biological results on the formation of newly built diatom valves support the high growth rates at salinities between 1 and 30 S_A_. Already at the first sampling after 3 days, almost every valve was found as newly formed. Further, due to partly reaching the stationary phase within these days, in combination with clumping, less valves were formed after 7 days resulting in partly higher unstained valves than those that were found on day three. Therefore, a clear distinction of a significant influence of the treatment after 3 days of growth is impossible. These results were similar to valve formation in dependence of temperature. The temperature of the respective four study sites seasonally ranged from 4.0 to 21.9°C for the Baltic Sea and from 4.2 to 31.8°C for the peatlands (data from 2019). During sampling in June, the Baltic Sea temperature was 19.7°C and that of the Hütelmoor already 26.1°C. The maximum growth rate at 15–20°C of the four investigated strains was slightly higher than the average temperature in the respective habitat. In accordance with many other studies, cultivated diatoms tend to grow best at slightly higher temperatures compared to their natural habitat (Suzuki and Takahashi, 1995; Woelfel et al., 2014; Schlie and Karsten, 2017). For example, the occurrence of *N. filiformis* and *Melosira* spp. increased significantly at the discharge site of cooling water from a nuclear power plant which enhanced the water temperatures 6–10°C on average (Snoeijs, 2011).

The four benthic diatoms were able to grow well from 5 to 30°C with at least a 20% percentile of the growth rates, except for *N. filiformis*, which was not able to grow at 5°C. This coincides with the findings of Woelfel et al. (2014) on growth rates of coastal Baltic Sea benthic diatoms, which ranged from 7 to 27°C with at least a 20% percentile of the growth rates. Admiraal (1977) studied 3 marine benthic diatom species from the Netherlands and reported a growth response between 4 and 25°C with the highest rates at 25°C. In a similar approach Scholz and Liebezeit (2012) investigated even 25 benthic diatom isolates from the German North Sea coast, and found optimum growth between 10 and 30°C, with strong species-specific reduction or inhibition at 4°C. The annual temperature of the Baltic Sea rarely surpasses 20°C. In contrast, adjacent peatlands exhibit ∼30°C in summer, indicating that the origin of the respective diatom strains might be reflected in their growth rates, as the peatland diatoms are exposed to a much broader annual temperature range. However, no distinct differences in growth rates were found for the Baltic Sea and peatland diatom strains. This wide response can be explained as growth of diatoms is mainly impacted by physiological processes that are involved in photosynthesis, more specific in the uncoupling of C and N assimilation (Gleich et al., 2020; and references therein).

### Temperature-Dependent Photosynthesis and Respiration

All four strains exhibited an efficiency of at least 20% over a large span of temperatures, clearly displaying eurythermal traits for photosynthesis and respiration. The strains isolated from the peatlands, *Planothidium* sp. (st. 1), *Planothidium* sp. (st. 2), and *N. filiformis*, displayed photosynthetic optima at higher temperatures ranging from 20 to 35°C whereas the Baltic Sea isolate *M. nummuloides* had its highest oxygen production at 10°C. This is according to the expectations that peatland species have to cope with higher temperature amplitudes. The small water bodies within the peatland contain large amounts of humic substances and hence absorb high solar radiation, resulting in higher water temperatures in summer compared to the Baltic Sea. Therefore, peatland algae must be adapted to higher temperature. However, there are still species-specific optima found, as *N. filiformis* was the only strain showing positive oxygen production over the whole range of temperatures. Prelle et al. (2019) used the same methodical approach to study the temperature dependent oxygen production in eight benthic diatom species from the southern Baltic Sea. Similar to the present study, they found that the optimum temperature for photosynthesis was lower compared to respiration. For diatoms, but also for green algae, temperature dependence of respiration and photosynthesis differs, as photosynthesis is more dependent on light, while respiration is mainly controlled by temperature dependent enzymatic activity (Atkin and Tjoelker, 2003; Karsten et al., 2016). This is partly confirmed in a more recent paper by Gleich et al. (2020), in which the authors indicate that light-dependent photosynthetic reactions are rather unaffected by temperature, while the carbon fixation reactions are not. In addition, a strong coupling of respiratory and photosynthetic activities is found in diatoms, which is explained by very tight physical interactions between chloroplasts and mitochondria (Bailleul et al., 2015). As a consequence, respiration is stimulated by light which results in an optimum ATP/NADPH ratio for subsequent carbon fixation (Bailleul et al., 2015).

### Light-Dependent Photosynthesis

Photosynthesis is primarily dependent on light availability. The PI-curves revealed species-specific photosynthetic activity with highest NPP_max_ for *N. filiformis* (106.94 μmol O_2_ mg^–1^ chl *a* h^–1^) and lowest for *Melosira nummuloides* (28.02 μmol O_2_ mg^–1^ chl *a* h^–1^). A previous study estimated a similar NPP_max_ of 23.12 μmol O_2_ mg^–1^ chl *a* h^–1^ for a benthic *Melosira*-species from the Baltic Sea (Prelle et al., 2019). Additionally, lowest dark respiration rates as reported by Prelle et al. (2019) were similar to dark respiration rates of this study with *N. filiformis* exhibiting lowest rates.

For *N. filiformis* and *Planothidium* sp. (st. 2) a discrepancy in the absolute photosynthetic values at the same temperature between light-dependent and temperature-dependent photosynthesis was noticeable. While self-shading was avoided using always low cell numbers, the methodological approach of the temperature-dependent photosynthesis might explain such differences as the initial experimental temperature of 5°C strongly diverged from culture conditions (20°C). Therefore, a possible initial temperature shock is assumed, which lowered photosynthetic performance during measurements. While there was no temperature change during the light-dependent photosynthesis, strains exhibited higher photosynthetic activity.

Low light compensation points of 14.1–21.9 μmol photons m^–2^ s^–1^ in combination with low light saturation points of 29.8–72.6 μmol photons m^–2^ s^–1^ for all four isolates may indicate low light requirements for photosynthesis. The peatland diatoms are often exposed to high amounts of humic substances in their habitats, which lead to enhanced turbidity and hence shading in the small water bodies and therefore face low light availability. In contrast, the Baltic Sea is a highly dynamic environment concerning the light field. Besides meteorological conditions and seasonality, wind-induced waves and bioturbation can lead to temporal burial of benthic diatom cells, being faced with darkness inside the sediment. Vertical movement of raphid species can overcome such conditions. Moreover, *M. nummuloides* has been reported to inhabit organically polluted parts of the Clyde Estuary in Scotland, which can be deoxygenated with an increased content of suspended soil particles leading to high water turbidity. This isolate can live in depths exceeding the light penetration level at high tides (McLean et al., 1981) and therefore is able to tolerate low light climate similar to our peatland strains. While the low to moderate light availability in their natural habitat is reflected in low light requirements for photosynthesis in all four strains, it can also be a result of the low light conditions of 30–50 μmol photons m^–2^ s^–1^ during cultivation. Although Serôdio and Lavaud (2011) showed that light requirements for photosynthesis are influenced by the laboratory growth conditions and hence might be the result of an acclimation to the culture maintenance, benthic diatoms are well known for their high photo-physiological plasticity (Ezequiel et al., 2015). Physiological and behavioral photoprotection (vertical movement into or out of the sediment) are the key mechanisms by which natural microphytobenthic communities protect themselves against high incident solar radiation (Cartaxana et al., 2011). In addition, benthic diatoms can quickly adjust their photosynthetic apparatus to new light conditions (Glud et al., 2002), for example, by alterations of the size or composition of the photosynthetic units (Richardson et al., 1983). All PI-curves showed light saturation and three out of the four cultures exhibited–partly minor–photoinhibition at 1,500 μmol photons m^–2^ s^–1^. This is similar to the findings of Prelle et al. (2019) as only minor photoinhibition was reported in three out of eight benthic diatom species. However, a considerable decrease in photosynthetic activity was found in *M. nummuloides* above 1,300 μmol photons m^–2^ s^–1^. In diatoms, excessive light can be dissipated by non-photochemical quenching via the de-epoxidation state of the xanthophyll cycle (Serôdio et al., 2008). Further absorption of excessive light by the light harvesting complexes can lead to photoinhibition, the decrease of the photosynthetic efficiency, that cannot be safely dissipated as heat (non- photchemical quenching) and thereby lowering the quantum yield (Goss and Lepetit, 2015). Even though we cannot prove the underlying mechanism of the observed photoinhibition in the investigated species, only few explanations exist. One could be either due to damage of the photosynthetic apparatus (D1 protein of PSII), or because of a regulated and reversible process comprising the xanthophyll cycle (Goss and Lepetit, 2015) as response to > 1,300 photons m^–2^ s^–1^, which still underline the high photosynthetic plasticity of benthic diatoms from the Baltic Sea coast.

### Lipid Droplets

Under stress diatoms are known to store intracellular oil droplets through carbon fixation as reserve material during vegetative growth (D’Ippolito et al., 2015), however, the mechanism of high lipid production remains unclear (Sayanova et al., 2017). While the average lipid content of an oleaginous diatom is about 23% of the dry cell weight under habitual growth conditions, it can increase up to 45% dry cell weight under stress (Hu et al., 2008). In general, temperature effects on lipid volume were rather small in the four benthic diatom strains, as the first sampling did not exhibit significant difference in lipid droplet volume. Over time, the lipid droplet volume increased in most strains at 17 and 22°C due to a shift from exponential to stationary growth phase. Significant differences by the treatment in the stationary phase at day 7 were only found in *M. nummuloides* and *Planothidium* sp. (st. 2) at 17°C. Therefore, lower salinity represents unfavorable growth conditions for *M. nummuloides* that consequently should lead to a higher lipid accumulation at stationary phase. The number of lipid droplets per cell was not affected. The extent to which TAGs are produced is species- and strain-specific (Hu et al., 2008), as not every species is able to produce and accumulate TAGs in high amounts. Zhang et al. (2020) studied highly oleaginous diatom strains adapted to high temperatures. While the highest TAG content was obtained at 246.4 mg/g dry biomass (*Fistulifera* sp. HB236), the lowest TAG content was half as much (105.9 mg/g dry biomass; *Nitzschia palea* HB170). Diatoms from habitats with fluctuating environmental conditions have been shown to build up larger lipid storages to ensure their survival (Van Duong et al., 2015). Diatoms can adapt to numerous stressors with broad tolerances toward salinity, temperature and light availability as previously mentioned. Therefore, it is likely that the temperature range in which lipid volume variations were studied was too narrow to induce a rapid build-up of large energy stores. Temperature effects on the lipid volume of the individual strains were investigated at temperatures (7, 17, and 22°C) at which all strains exhibited growth above 45–85% percentile of the optimum growth rates as well as positive oxygen production rates. Based on the growth rates and their wide temperature tolerance it may be assumed that the experimental conditions, under which temperature effects on lipid volume have been studied, did not lead to stress.

Analogous to temperature fluctuations, strong salinity fluctuations in the habitat may lead to the production and storage of biochemical energy reserves in microalgae in larger quantities than necessary under constant conditions (Lim et al., 2012; Van Duong et al., 2015). Results of *N. filiformis* suggest a salinity effect as lipid volume decreased significantly with increasing salinity. In contrast, the growth rates of this strain remained constant at salinities ranging from 1 to 39 S_A_ with no significant effect. Therefore, degradation of lipid storage as a stress response may prevent a decrease in growth at higher salinities. Interpretations have to be treated with caution, as these observations are limited due to the small number of evaluable cells. *Melosira nummuloides* did not grow at 1 S_A_ but grew well at salinities from 5 to 39 S_A_, with no significant differences at increasing salinities. Therefore, lower salinity represents unfavorable growth conditions for *M. nummuloides* that consequently should lead to a higher lipid accumulation at stationary phase. However, our results cannot confirm this assumption. At the second sampling, lipid volume of *M. nummuloides* at 1 S_A_ exhibited the lowest volume compared to the higher salinities. A possible explanation is, that low salinities from the experimental and pre-culture phase constitute high stress levels over a certain period of time entailing that this strain was unable to build-up reserve lipids or even started to break down the lipid stores already. At the second sampling *Planothidium* sp. (st. 1) exhibited highest growth rates and an increase in lipid volume under marine conditions. Furthermore, an increase in lipid content for *Planothidium* sp. (st. 2) was observed under optimal growth conditions, which indicates the lipid production to a certain homeostasis also at non-stress, fast growth conditions. It is therefore questionable if lipid volume is a good indicator for stress responses in benthic diatoms and hence multiple experimental approaches are required for clear results.

## Conclusion

In conclusion, all four benthic diatom strains exhibited euryhaline and eurythermal growth responses surpassing the annual fluctuations of the respective habitats. The optimum temperature for photosynthesis of the Baltic Sea diatom strain was at 10°C and those of the peatland diatoms at 20–35°C, reflecting the environmental temperature of the respective habitats. All strains exhibited low-light requirements, which is of advantage in those habitats with high turbidity. The lipid content as a potential stress marker did not show clear results, indicating less suitability as previously thought. Overall, due to their eurythermal and euryhaline traits along with high photo-physiological plasticity, all four benthic diatom strains seem well adapted to cope and survive highly fluctuating abiotic parameters and gradients in the sea-land transition zone of the southern Baltic Sea coast.

## Data Availability Statement

The datasets presented in this study can be found in online repositories. The names of the repository/repositories and accession number(s) can be found in the article/Supplementary Material.

## Author Contributions

LP, MA, UK, and KG developed the idea and elaborated the concept. LP, MA, PD, TG, JJ, SM, LS, LV, and KG provided experimental and taxonomic data. All authors organized and conducted the data analyses, involved in writing the first draft of the manuscript, which was commented and edited by LP, MA, UK, and KG and finally accepted by all authors.

## Conflict of Interest

The authors declare that the research was conducted in the absence of any commercial or financial relationships that could be construed as a potential conflict of interest.

## Footnotes

1 www.algaebase.org
