# Supplementary material for: Ecophysiological and Cell Biological Traits of Benthic Diatoms From Coastal Wetlands of the Southern Baltic Sea
Source: Front Microbiol. 2021 Apr 12;12:642811. doi: 10.3389/fmicb.2021.642811 (PMC8072133; doi:10.3389/fmicb.2021.642811)
Supplement: Supplementary file 1 [file Table_1.doc]

**Supplementary Table 1**. Confidence intervals for temperature-dependent growth rates.

|  | 2.5% | 97.5% |
| --- | --- | --- |
| ***Nitzschia filiformis*** |  |  |
| maximal growth rate | 0.749 | 0.970 |
| optimum growth temperature | 18.526 | 21.734 |
| maximum growth temperature | 30.537 | 34.189 |
| maximal photosynthetic rate | 47.374 | 60.930 |
| optimum photosynthetic temperature | 21.957 | 26.405 |
| maximum photosynthetic temperature | 40.000 | 45.459 |
| maximum respirational rate | -34.759 | -26.470 |
| optimum respirational temperature | 32.987 | 36.133 |
| maximum respirational temperature | 41.754 | 48.939 |
| ***Planothidium sp.* (st. 1)** |  |  |
| maximal growth rate | 1.018 | 1.208 |
| optimum growth temperature | 18.706 | 20.973 |
| maximum growth temperature | 31.580 | 34.612 |
| maximal photosynthetic rate | 42.389 | 60.713 |
| optimum photosynthetic temperature | 26.013 | 30.383 |
| maximum photosynthetic temperature | 38.522 | 41.324 |
| maximum respirational rate | -46.481 | -37.961 |
| optimum respirational temperature | 28.073 | 41.036 |
| maximum respirational temperature | 40.967 | 77.819 |
| ***Planothidium sp.* (st. 2)** |  |  |
| maximal growth rate | 1.074 | 1.198 |
| optimum growth temperature | 14.996 | 16.912 |
| maximum growth temperature | 32.460 | 35.205 |
| maximal photosynthetic rate | 18.639 | 26.084 |
| optimum photosynthetic temperature | 19.153 | 24.537 |
| maximum photosynthetic temperature | 35.307 | 37.968 |
| maximum respirational rate | -52.748 | -42.895 |
| optimum respirational temperature | 31.020 | 33.339 |
| maximum respirational temperature | 42.209 | 46.708 |
| ***Melosira nummuloides*** |  |  |
| maximal growth rate | 1.058 | 1.209 |
| optimum growth temperature | 16.819 | 18.930 |
| Maximum growth temperature | 32.281 | 35.364 |
| maximal photosynthetic rate | 34.952 | 63.164 |
| optimum photosynthetic temperature | 0.341 | 17.808 |
| maximum photosynthetic temperature | 30.773 | 39.828 |
| maximum respirational rate | -20.935 | -13.124 |
| optimum respirational temperature | 29.478 | 40.193 |
| maximum respirational temperature | 35.731 | 62.395 |
